# Diagnostic value of clusterin immunostaining in hepatocellular carcinoma

**DOI:** 10.1186/s13000-020-01041-8

**Published:** 2020-10-14

**Authors:** Yuan Li, Fangfang Liu, Wendi Zhou, Sharon Zhang, Peiguo Chu, Fan Lin, Hanlin L. Wang

**Affiliations:** 1 Department of Pathology and Laboratory Medicine, David Geffen School of Medicine at UCLA, 10833 Le Conte Avenue, Los Angeles, CA 90095 USA; 2 Department of Pathology, Molecular Pathology Research Center, Peking Union Medical College Hospital, Chinese Academy of Medical Science, Beijing, 100730 China; 3 Department of Pathology, Peking University People’s Hospital, Xicheng District, Beijing, 100044 China; 4 Present address: Department of Pathology, Banner University Medical Center, Phoenix, AZ 85006 USA; 5 Present address: Department of Pathology, Adventist Health, Hanford, CA 93230 USA; 6 Department of Pathology, City of Hope National Medical Center, Duarte, CA 91010 USA; 7 Present address: Anatomic Pathology, NeoGenomics Laboratories, California, USA; 8 Department of Laboratory Medicine, Geisinger Medical Center, Danville, PA 17822 USA

**Keywords:** Clusterin, pCEA, CD10, Hepatocellular carcinoma, Immunohistochemistry

## Abstract

**Background:**

Histologic distinction between well differentiated hepatocellular carcinoma (HCC) and benign hepatocellular mass lesions is a known challenge. Existing biomarkers are of limited diagnostic value. Our previous studies observed an enhanced canalicular expression pattern of clusterin (CLU) in HCC, which was not observed in benign hepatocellular mass lesions such as hepatocellular adenoma. The aim of this study was to further investigate its diagnostic value for HCC by examining the expression pattern of CLU in a large number of non-hepatocellular tumors, and by comparing it with two other commonly used hepatocellular markers pCEA and CD10 that also show a canalicular staining pattern in HCC.

**Methods:**

Enhanced canalicular staining patterns of CLU, pCEA and CD10 were analyzed on 54 surgically resected well to moderately differentiated HCCs on whole tissue sections, of which 37 had surrounding regenerative nodules while the remaining 17 had a non-cirrhotic background. CLU immunostaining was also performed on tissue microarray sections that contained 74 HCCs (40 of which were also stained for pCEA and CD10), 55 normal liver tissue samples, and 1305 non-hepatocellular tumors from multiple organs.

**Results:**

Enhanced CLU canalicular staining was observed in 70% (89/128) HCCs but not in regenerative nodules, normal liver tissues or any non-hepatocellular tumors. The sensitivity and specificity for enhanced canalicular staining pattern of CLU in HCCs were 0.70 and 1.00. This enhanced canalicular pattern was observed in only 26 and 23% HCCs for CD10 and pCEA, respectively. These results further demonstrate that the distinctive enhanced canalicular pattern of CLU is unique to HCC.

**Conclusions:**

CLU is superior to pCEA and CD10 as a diagnostic immunomarker in that it can help distinguish well to moderately differentiated HCC not only from non-HCC malignancies but also from benign hepatocellular mass lesions.

## Introduction

Clusterin (CLU) is a highly conservative multifunctional glycoprotein present in almost all types of mammalian tissue and most human body fluids [1]. Its high degree of conservation and its wide tissue distribution suggest that it plays a fundamental biological role. There are two proteins encoded by the *CLU* gene: secretory CLU protein (sCLU) and nuclear CLU protein (nCLU). It has been recognized that sCLU, also known as apolipoprotein J (ApoJ), is an important extracellular chaperone involved in a broad range of physiological and pathophysiological processes, including tissue remodeling, reproduction, lipid transport, complement regulation, and programed cell death [2]. CLU has been shown to be overexpressed in several human cancers, such as carcinomas of the prostate [3, 4], breast [5, 6], colon [7], and lung [8]. Overexpression of CLU has been correlated with increased tumor aggressiveness, chemotherapy and radiotherapy resistance, and poor prognosis [9–11].

We have previously observed an enhanced canalicular CLU expression pattern in hepatocellular carcinoma (HCC) by immunohistochemistry, which has the diagnostic potential to help distinguish HCC from benign hepatocellular mass lesions [12]. However, the diagnostic value of this unique staining pattern to distinguish HCC from non-hepatocellular tumors has not been studied. Furthermore, the CLU staining pattern in HCC is somewhat similar to those demonstrated by polyclonal CEA (pCEA) and CD10. These latter two immunomarkers may help determine hepatocellular origin, but do not appear to distinguish between benign and malignant hepatocellular mass lesions.

The aim of this study was to examine the distribution and pattern of CLU expression in tumors of various origins to further investigate the diagnostic value of enhanced canalicular staining pattern for HCC. We also compared the expression pattern of CLU with those of pCEA and CD10 in HCC and its surrounding nonneoplastic liver tissue.

## Materials and methods

### Specimens

Tissue blocks selected from 54 surgically resected HCCs were used to compare the immunohistochemical staining patterns of CLU, pCEA and CD10 on whole tissue sections. Thirty-seven of these cases had surrounding background liver which was cirrhotic with regenerative nodules (RNs). The remaining 17 cases had a non-cirrhotic background. CLU immunostaining was also performed on tissue microarray (TMA) sections containing 74 HCCs and 55 normal liver tissue samples. Forty HCCs on TMA sections were also stained for pCEA and CD10. The HCC cases on TMA sections did not include adjacent nonneoplastic liver tissues. All enrolled HCCs were well to moderately differentiated. None of them had been treated with neoadjuvant chemotherapy or embolization. In addition, 1305 tumor samples from multiple organs on TMAs were used to detect CLU expression. These included esophageal adenocarcinoma (*n* = 48), colorectal adenocarcinoma (*n* = 86), pancreatic ductal adenocarcinoma (*n* = 48), pancreatic neuroendocrine tumor (*n* = 14), cholangiocarcinoma (*n* = 13), lung adenocarcinoma (*n* = 97), lung squamous cell carcinoma (*n* = 74), breast ductal adenocarcinoma (*n* = 86), papillary thyroid carcinoma (*n* = 48), prostatic adenocarcinoma (*n* = 96), clear cell renal cell carcinoma (*n* = 78), papillary renal cell carcinoma (*n* = 33), adrenocortical tumor (*n* = 30), urothelial carcinoma (*n* = 78), uterine endometrioid carcinoma (*n* = 93), ovarian serous carcinoma (*n* = 40), endocervical adenocarcinoma (*n* = 37), clear cell carcinoma of the uterus and ovary (*n* = 28), germ cell tumors (*n* = 184), mesothelioma (*n* = 31), squamous cell carcinoma of the head and neck (*n* = 49), and perivascular epithelioid cell tumor (PEComa or epithelioid angiomyolipoma; *n* = 14). TMAs were constructed as previously described [13].

### Immunohistochemistry

Formalin-fixed, paraffin-embedded tissue sections (whole sections and TMAs) were immunohistochemically stained for CLU using the DAKO autostainer following the manufacturer’s instructions. Briefly, deparaffinized 5-μm sections were rehydrated and treated with 3% hydrogen peroxide for 15 min. Following heat-induced epitope retrieval in 10 mmol/L citrate buffer (pH 6.0), the tissue sections were incubated with a purified mouse anti-human clusterin monoclonal antibody (clone E5, BD Biosciences, San Jose, CA, United States) used at 1:40 dilution for 1 h at room temperature. The immunoreaction was developed using the EnVision+ detection system that contained biotin-free horseradish peroxidase-labelled polymers (DAKO, Carpinteria, CA, United States). The staining was visualized using 3,3′-diaminobenzidine substrate-chromogen solution and counterstained with hematoxylin. In each experiment, a negative control was included in which the primary antibody was replaced by non-human-reactive mouse IgG.

Whole tissue sections that contained HCC and surrounding nonneoplastic liver tissue were also stained for CD10 using a prediluted rabbit monoclonal antibody SP67 following cell conditioning 1 (CC1) mild antigen retrieval and for pCEA using a rabbit polyclonal antibody used at 1:200 dilution following CC1 antigen retrieval. Both CD10 and pCEA immunostains were performed using the Ventana BenchMark Ultra system (Indianapolis, IN, United States). The incubation time for primary antibodies was 12 min and 20 min for CD10 and pCEA, respectively.

Canalicular staining patterns of CLU, pCEA and CD10 were analyzed on immunostained slides. A case was recorded as positive if ≥10% of tumor cells expressed canalicular immunoreactivity. A case was considered negative if canalicular immunoreactivity was observed in < 10% of tumor cells. For HCC cases, the staining intensity was compared between tumor and surrounding nonneoplastic liver tissue to determine if the canalicular immunoreactivity in HCC was enhanced (exaggerated canalicular pattern or stronger staining intensity along the canalicular spaces between tumor cells in comparison with surrounding nonneoplastic hepatocytes), equivalent or weaker. “Luminal” immunoreactivity was also considered as canalicular pattern for HCC cases with prominent pseudoglandular/pseudoacinar formation.

### Statistical analysis

The canalicular staining patterns were compared among CLU, CD10, and pCEA in HCC cases. Statistical analysis was performed by Pearson Chi-square tests using the SPSS version 23. A *P*-value < 0.05 was considered statistically significant.

## Results

### Enhanced CLU canalicular staining pattern was observed in majority of HCCs but not in nonneoplastic liver tissue

**Fig. 1:**
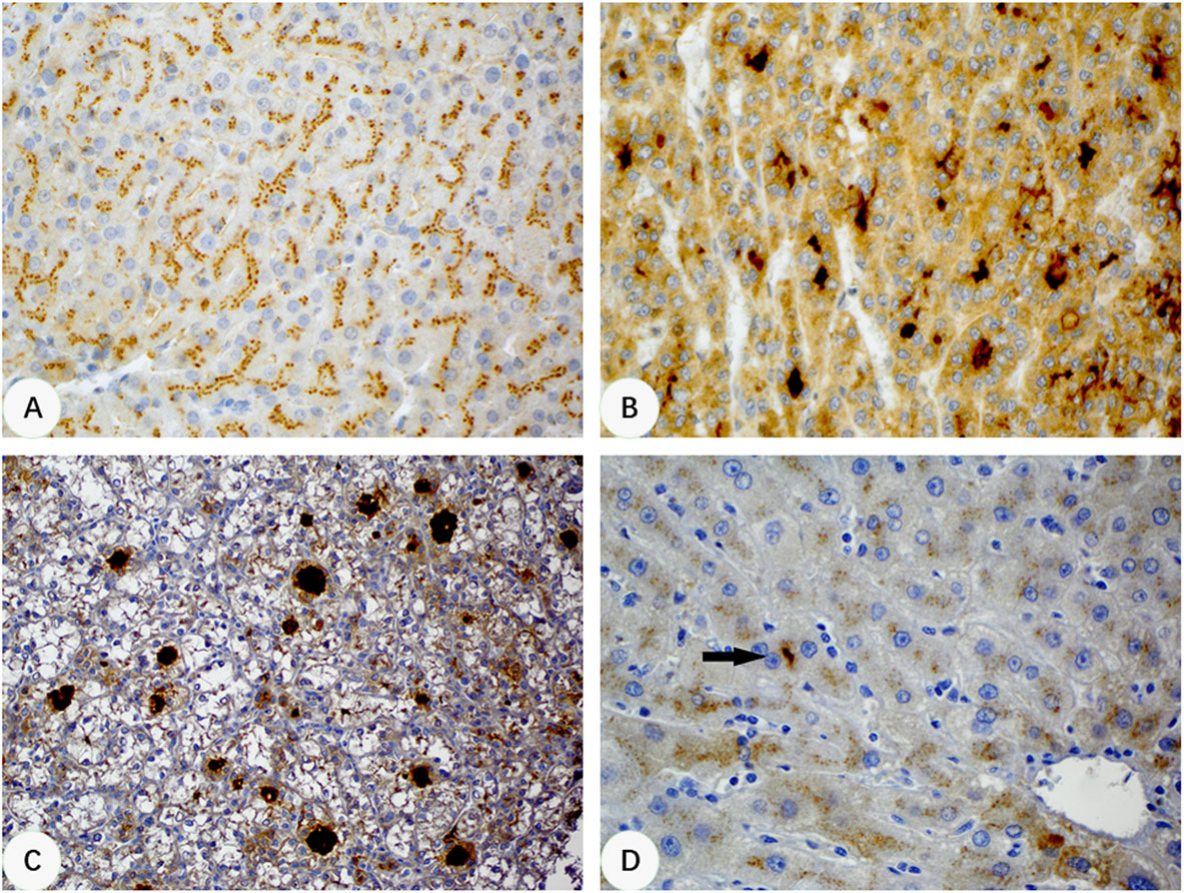
Immunohistochemical staining patterns of CLU in normal liver, RNs and HCC. **a**, Normal liver tissue showing a delicate, fine granular and “railroad track”-like pericanalicular pattern (400x). **b**, A HCC showing a unique enhanced canalicular staining pattern (400x). **c**, A HCC with pseudoglandular structures showing intraluminal staining pattern (200x). D, A RN showing focal enhanced canalicular staining pattern involving one canaliculus (arrow, 400x)

In normal and cirrhotic liver tissues, CLU immunostaining highlighted intercellular canaliculi with a delicate, fine granular and “railroad track”-like pericanalicular pattern (Fig. 1a). However, this “benign” pattern had changed when there was a malignant transformation. In HCCs, a much enhanced and exaggerated canalicular staining pattern (Fig. 1b) was observed in 89 of 128 (70%) HCCs. This included cases with pseudoglandular/pseudoacinar formation that exhibited an intraluminal staining pattern (Fig. 1c). Of the 54 surgically resected HCCs where whole tissue sections were used for the study, 42 (77.8%) showed enhanced canalicular CLU staining pattern. Among them, 10 (18.5%) were positive in > 50% of tumor cells (diffuse), 20 (37%) in 26–50% of tumor cells (patchy), and 12 (22.2%) in 10–25% of tumor cells (focal). There was no significant difference in aberrant CLU expression between well and moderately differentiated HCCs, seen in 10 of 14 (71.4%) and 32 of 40 (80%) cases, respectively. None of the 55 normal liver tissue samples on TMA and none of 17 non-cirrhotic background liver tissues on whole tissue sections showed this enhanced staining pattern. In 37 cases with a cirrhotic background on whole tissue sections, 20 (54%) showed focal enhanced canalicular pattern of CLU in RNs, but positive cells in all these cases were < 10% (thus considered negative), usually < 3%, involving only one or a few canaliculi (Fig. 1d). The difference between HCC and RN was statistically significant (*p* < 0.001) (Table 1). The sensitivity and specificity for enhanced canalicular staining pattern of CLU in HCCs were 0.70 and 1.00, respectively. Other expression patterns of CLU observed in HCCs included cytoplasmic (without canalicular staining), paranuclear dot-like, and membranous staining, seen in 6, 4 and 3 cases, respectively.

**Table 1:** Comparison of CLU, CD10, pCEA immunoreactivity between HCC and RN

Antigen	Enhanced canalicular pattern no. (%) positive		*P* value
	HCC	RN	
CLU			
> 10%	89 (70)	0	< 0.001
< 10%	4 (3)	20 (54)	
Negative	35 (27)	17 (46)	
Total	128	37	
CD10			
> 10%	24 (26)	0	0.002
< 10%	6 (6)	5 (14)	
Negative	64 (68)	32 (86)	
Total	94	37	
pCEA			
> 10%	22 (24)	0	0.003
< 10%	4 (4)	4 (11)	
Negative	68 (72)	33 (89)	
Total	94	37	

*CLU* Clusterin, *pCEA* Polyclonal antibody against carcinoembryonic antigen, *HCC* Hepatocellular carcinoma, *RN* Regenerative nodule

### Enhanced CLU canalicular staining pattern was not observed in non-hepatocellular tumors

**Fig. 2:**
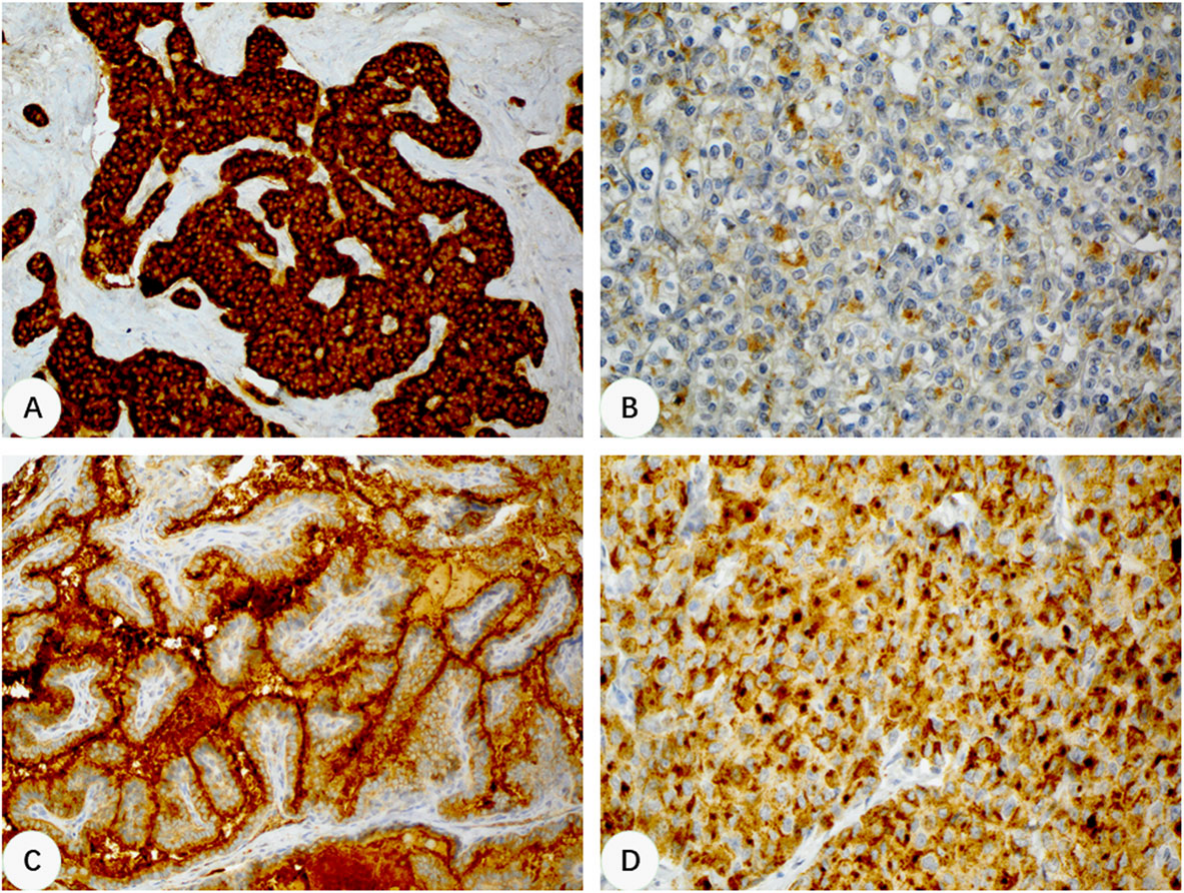
Immunohistochemical staining patterns of CLU in non-hepatocellular tumors of various origins. **a**, A pancreatic neuroendocrine tumor showing diffusely strong cytoplasmic and membranous staining (200x). **b**, A clear cell renal cell carcinoma showing patchy weak cytoplasmic positivity (400x). **c**, A papillary thyroid carcinoma showing luminal surface staining (200x). **d**, A breast ductal carcinoma showing cytoplasmic and paranuclear dot-like staining (400x)

Three CLU staining patterns were observed in non-hepatocellular tumors: cytoplasmic with (Fig. 2a) or without (Fig. 2b) membranous staining, luminal/apical staining (Fig. 2c), and paranuclear dot-like staining (Fig. 2d). Cytoplasmic pattern was most common, which was seen in almost all tumor types. Among them, pancreatic neuroendocrine tumor showed strong and diffuse cytoplasmic and membranous expression in 12 of 14 (86%) cases. A small fraction (4/48, 8.3%) of pancreatic ductal adenocarcinoma also showed strong cytoplasmic expression. Cytoplasmic and membranous staining was seen in cholangiocarcinoma (10/13, 76.9%), and PEComa (7/14, 50%). Weak cytoplasmic staining with or without paranuclear dot pattern was seen in lung adenocarcinoma (30/97, 30.9%), breast ductal adenocarcinoma (75/86, 87.2%), clear cell renal cell carcinoma (67/78, 85.9%), clear cell carcinoma of the uterus and ovary (22/28, 78.6%), adrenocortical tumor (22/30, 73.3%) and germ cell tumors (127/184, 69.0%). Luminal/apical staining pattern was commonly seen in tumors that had glandular or papillary structures, such as esophageal adenocarcinoma (15/48, 31.3%), papillary thyroid carcinoma (39/48, 81.3%), prostatic adenocarcinoma (18/96, 18.8%), endometrioid carcinoma of the uterus (41/93, 44.1%), serous carcinoma of the ovary (10/40, 25.0%). Colorectal carcinoma (6/86, 7.0%) and mesothelioma (4/31, 12.9%) showed the least CLU positivity. None of the non-hepatocellular tumors showed enhanced canalicular CLU staining pattern.

### CD10 and pCEA also showed an enhanced canalicular pattern in HCC but with a much lower frequency

**Fig. 3:**
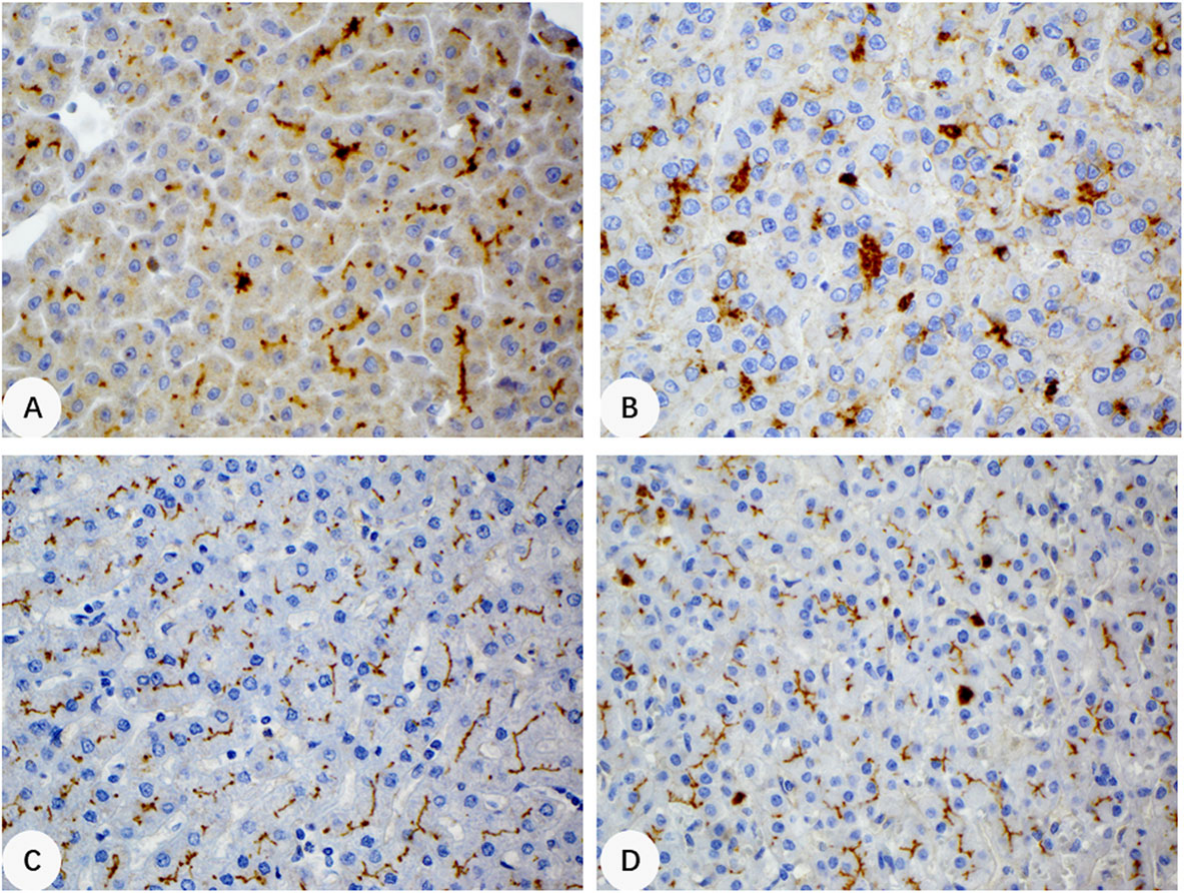
Immunohistochemical staining patterns of CD10 and pCEA. **a**, Enhanced CD10 canalicular staining seen in a HCC (400x). **b**, Enhanced pCEA canalicular staining in a HCC (400x). **c**, Linear canalicular staining pattern for CD10 in nonneoplastic liver tissue (400x). **d**, Linear canalicular staining pattern for pCEA in nonneoplastic liver tissue (400x). Positive staining was also noted in scattered inflammatory cells for pCEA

Positive canalicular staining for CD10 and pCEA was observed in 50 (53%) and 68 (72%) of 94 HCCs examined for these immunomarkers (Fig. 3a and b). Though more sensitive for the canalicular pattern than CD10, pCEA also showed cytoplasmic staining and positive staining in inflammatory cells, giving rise to a higher degree of background staining. The majority of CD10 and pCEA positive cases showed equivalent staining intensity between HCC and surrounding benign liver tissue (Fig. 3c and d). Enhanced canalicular pattern was observed in only 24 (26%) and 22 (23%) HCCs for CD10 and pCEA, respectively (Table 1). The sensitivity and specificity for enhanced canalicular staining pattern in HCC were 0.26 and 1.000 for CD10, and 0.23 and 1.000 for pCEA, respectively. Of the 54 surgically resected HCCs where whole tissue sections were used for the study, 4 (7.4%) were negative for all 3 markers. Eight cases (14.8%) were positive for CLU, but negative for both CD10 and pCEA. Eleven cases (20.4%) were positive for only one marker, either CLU, CD10 or pCEA.

## Discussion

Histologic distinction between well differentiated HCC and benign hepatocellular mass lesions such as hepatocellular adenoma (HCA), dysplastic nodule and RN is a known challenge to pathologists, especially on biopsy specimens. In addition to reticulin stain, the currently available immunomarkers that may help the distinction include glypican-3, glutamine synthetase, heat shock protein 70, CD34, and alpha-fetoprotein. Another diagnostic challenge that pathologists often face is to differentiate HCC from non-hepatocellular tumors that may be liver primaries or hepatic metastases. Diagnostic markers that may help in this regard include hepatocyte antigen (hepar-1), arginase-1, pCEA, CD10, and albumin. However, many of these markers suffer from low sensitivity and specificity, which has significantly limited their utility in clinical practice [14]. For example, glypican-3 is often negative in well differentiated HCC, but frequently positive in germ cell tumors. Focal positivity can also be detected in cirrhotic nodules [15, 16].

CLU is a multifunctional molecule that has been implicated in tumorigenesis and tumor progression. Kang et al. [17] examined CLU expression in 100 surgically resected HCCs and observed two distinct staining patterns: cytoplasmic and canalicular. Canalicular staining pattern was found in 71% of their cases, among which 17% also showed cytoplasmic staining. Interestingly, cases with a canalicular CLU pattern was found to be associated with an overall better prognosis than those with cytoplasmic or negative CLU staining. In another study [18], overexpression of CLU was found to promote cell migration and metastasis in HCC cell lines. Our previous studies not only found overexpression of CLU in hepatocellular neoplasms such as HCC and HCA, but also demonstrated a distinctive enhanced canalicular staining pattern exclusively seen in HCC [12, 19]. In one of these studies, 134 surgically resected HCCs were immunohistochemically examined for CLU expression. Overall, the enhanced canalicular CLU staining pattern was observed in 101 (75.3%) cases. The frequencies of this staining pattern were comparable between well and moderately differentiated HCCs, seen in 48 of 62 (77.4%) and 45 of 56 (80.4%) cases, respectively, but lower in poorly differentiated HCCs, seen in 8 of 16 (50%) cases. This study also examined 33 HCAs, 40 focal nodular hyperplasias and 77 large RNs. None of these benign hepatocellular mass lesions showed this “malignant” enhanced canalicular staining pattern [12].

In the present study, we first confirmed a similar frequency of enhanced canalicular CLU staining pattern in well and moderately differentiated HCCs. This pattern was not observed in normal liver tissue samples and nonneoplastic, noncirrhotic liver tissue surrounding HCC. However, this “malignant” pattern could be observed in RNs surrounding HCC on careful examination, but in very limited area usually involving only one or a few canaliculi. It is thus important to use 10% as a cut-off if CLU is to be used as a diagnostic immunomarker for HCC. Second, we demonstrated that enhanced canalicular CLU pattern was exclusive to HCC and was not observed in various tumors of non-hepatocellular origin. Third, we observed a similar enhanced canalicular staining pattern for pCEA and CD10 in HCC but with a much lower frequency in comparison with CLU.

Pseudoglandular or pseudoacinar structures are common in HCC, which can show a luminal CLU staining pattern. Interestingly, a luminal/apical CLU staining pattern was also demonstrated in some gland-forming carcinomas. However, the luminal staining in HCC is typically focal in contrast to the more diffuse pattern in most gland-forming carcinomas. Nevertheless, the distinction between HCC and these carcinomas is usually not a challenge on histologic grounds.

In nonneoplastic liver, CD10 and pCEA immunostains show a characteristic linear canalicular pattern, probably due to cross reactivity to biliary glycoprotein I present in bile canaliculi [20–22]. This pattern is retained in > 50% HCCs, which has been used to help confirm the hepatocellular origin in difficult cases. In this study, both CD10 and pCEA were found to show an enhanced canalicular pattern in a quarter of HCC cases, a much lower frequency in comparison with CLU. It is interesting to note that the CLU staining pattern switches from “railroad track”-like pericanalicular pattern in normal liver to enhanced canalicular pattern in HCC, while CD10 and pCEA maintain the similar canalicular pattern but slightly enhanced in a small subset of HCC. This significantly limits the diagnostic value of CD10 and pCEA in the differential diagnosis of benign and malignant hepatocellular lesions.

In summary, the data presented in this study extend our previous observations and further demonstrate that the distinctive enhanced canalicular pattern of CLU is unique to HCC and is not observed in non-hepatocellular tumors. Our data also demonstrate that CLU is superior to pCEA and CD10 as a diagnostic immunomarker in that it helps distinguish well to moderately differentiated HCC not only from non-HCC malignancies but also from benign hepatocellular mass lesions. The utility of CLU in the distinction between poorly differentiated HCC and non-hepatocellular malignancies is limited based on our previous studies because enhanced canalicular pattern is less commonly seen in poorly differentiated HCC. It remains to be investigated why a large subset of HCCs show enhanced canalicular CLU expression.

## Data Availability

The datasets used and/or analyzed during the current study are available from the corresponding author on reasonable request.

## Abbreviations

CLU: Clusterin
sCLU: Secretory clusterin protein
nCLU: Nuclear CLU protein
ApoJ: Apolipoprotein J
HCC: Hepatocellular carcinoma
pCEA: Polyclonal antibody against carcinoembryonic antigen
RN: Regenerative nodule
TMA: Tissue microarray
HCA: Hepatocellular adenoma
